# Muir Torre syndrome and MSH2 mutations: the importance of dermatological awareness

**DOI:** 10.1038/sj.bjc.6603228

**Published:** 2006-06-20

**Authors:** M Tischkowitz, A Gologan, H Srolovitz, M Khanna, W D Foulkes

**Affiliations:** 1 Cancer Prevention Centre, Sir MB Davis Jewish General Hospital, McGill University, Montreal, QC, Canada; 2 Department of Pathology, Sir MB Davis Jewish General Hospital, McGill University, Montreal, QC, Canada; 3 Division of Dermatology, Department of Medicine, Sir MB Davis Jewish General Hospital, McGill University, Montreal, QC, Canada

**Sir**,

We would like to give an update on a family with Lynch syndrome (hereditary non-polyposis colorectal cancer, HNPCC) that we have previously reported with germline truncating mutations in *MSH2* (exon 8 deletion) and BRCA2 (542G>T) (Thiffault *et al*, 2004). This was a 26-member kindred with five cases of colorectal cancer and five cases of breast cancer, all but one of the cancers occurring below the age of 45 years. We reported that one of the individuals who had been diagnosed with rectal cancer (III.10 in original pedigree) also had a keratoacanthoma. This type of skin lesion is seen in some families with *MSH2* or, less commonly, *MLH1* mutations when it is termed Muir–Torre Syndrome (MTS) (Ponti and Ponz de Leon, 2005). Immunohistochemistry (IHC) at the time showed normal MSH2 expression, so we felt that this family did not belong to the MTS group. Subsequently, the individual was diagnosed with two separate sebaceous carcinomas on each arm. Immunohistochemistry analysis of these lesions shows loss of *MSH2* expression in both cases, one of which is shown in Figure 1, confirming that this is in fact an MTS family.

This new development illustrates two points. Firstly the Lynch syndrome and MTS phenotypes are pleiotropic and Lynch syndrome can evolve into MTS in the same family. Lynch syndrome is usually suspected when the Amsterdam Criteria are fulfilled or the less-specific Bethesda guidelines are met. The individual described here had an anal canal squamous carcinoma, a cancer type not associated with Lynch syndrome, which was microsatellite stable. His father, who was an obligate MSH2 mutation carrier, had a rectal cancer which is also unusual (Hoogerbrugge *et al*, 2003), and an astrocytoma, raising the possibility of overlap with another variant, Turcot syndrome. Although Turcot syndrome was classically thought of as a combination of brain tumours and polyposis and has been mainly associated with mutations in the APC gene (Galiatsatos and Foulkes, 2006), a minority are also due to mutation in the Lynch syndrome genes, particularly biallelic PMS mutation carriers (De Vos *et al*, 2006). Secondly, the case emphasises the importance of continuing dermatological vigilance in *MSH2* families. The same *MSH2* mutations are found in both MTS and Lynch families (Ponti and Ponz de Leon, 2005), so it is not possible to predict which families are more likely to develop MTS. The sebaceous cancers in MTS are possibly less aggressive than sporadic types (Ponti and Ponz de Leon, 2005), but little is known about survival in MTS.

## Figures and Tables

**Figure 1:**
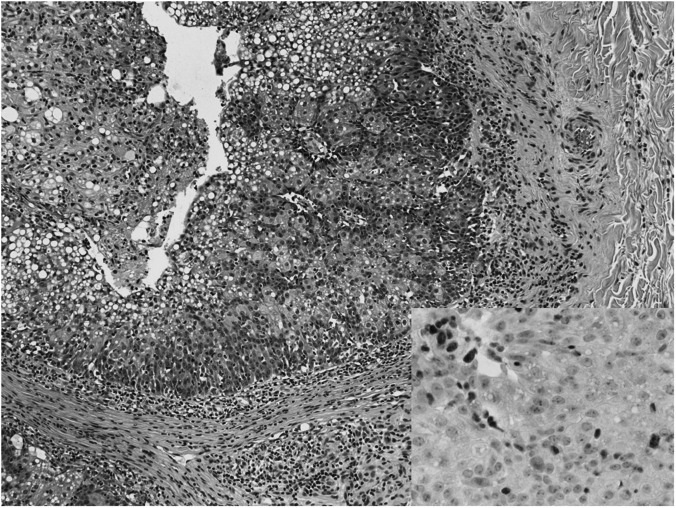
Sebaceous carcinoma: haematoxylin and eosin (× 100): multilobulated expansile intradermal tumour showing large polygonal cells with differentiation into sebaceous cells, altered nuclear/cytoplasmic ratio, evidence of apoptosis and high mitotic count. Insert: MSH2 immunohistochemical stain (× 400): Absence of nuclear staining for the MSH2 mismatch repair protein in the tumour cells (the tumour infiltrating lymphocytes show normal expression of MSH2).
